# A multi-dimensional framework for prosthetic embodiment: a perspective for translational research

**DOI:** 10.1186/s12984-022-01102-7

**Published:** 2022-11-11

**Authors:** Jan Zbinden, Eva Lendaro, Max Ortiz-Catalan

**Affiliations:** 1 Center for Bionics and Pain Research, Mölndal, Sweden; 2 Department of Electrical Engineering, Chalmers University of Technology, Gothenburg, Sweden; 3 Department of Brain and Cognitive Sciences, McGovern Institute for Brain Research, Massachusetts Institute of Technology, Cambridge, MA USA; 4 Operational Area 3, Sahlgrenska University Hospital, Gothenburg, Sweden; 5 Department of Orthopaedics, Institute of Clinical Sciences, Sahlgrenska Academy, University of Gothenburg, Gothenburg, Sweden

**Keywords:** Embodiment, Prosthetics, Ownership, Agency, Body representation, Phenomenology

## Abstract

The concept of embodiment has gained widespread popularity within prosthetics research. Embodiment has been claimed to be an indicator of the efficacy of sensory feedback and control strategies. Moreover, it has even been claimed to be necessary for prosthesis acceptance, albeit unfoundedly. Despite the popularity of the term, an actual consensus on how prosthetic embodiment should be used in an experimental framework has yet to be reached. The lack of consensus is in part due to terminological ambiguity and the lack of an exact definition of prosthetic embodiment itself. In a review published parallel to this article, we summarized the definitions of embodiment used in prosthetics literature and concluded that treating prosthetic embodiment as a combination of ownership and agency allows for embodiment to be quantified, and thus useful in translational research. Here, we review the potential mechanisms that give rise to ownership and agency considering temporal, spatial, and anatomical constraints. We then use this to propose a multi-dimensional framework where prosthetic embodiment arises within a spectrum dependent on the integration of volition and multi-sensory information as demanded by the degree of interaction with the environment. This framework allows for the different experimental paradigms on sensory feedback and prosthetic control to be placed in a common perspective. By considering that embodiment lays along a spectrum tied to the interactions with the environment, one can conclude that the embodiment of prosthetic devices should be assessed while operating in environments as close to daily life as possible for it to become relevant.

## Introduction

**Fig. 1:**
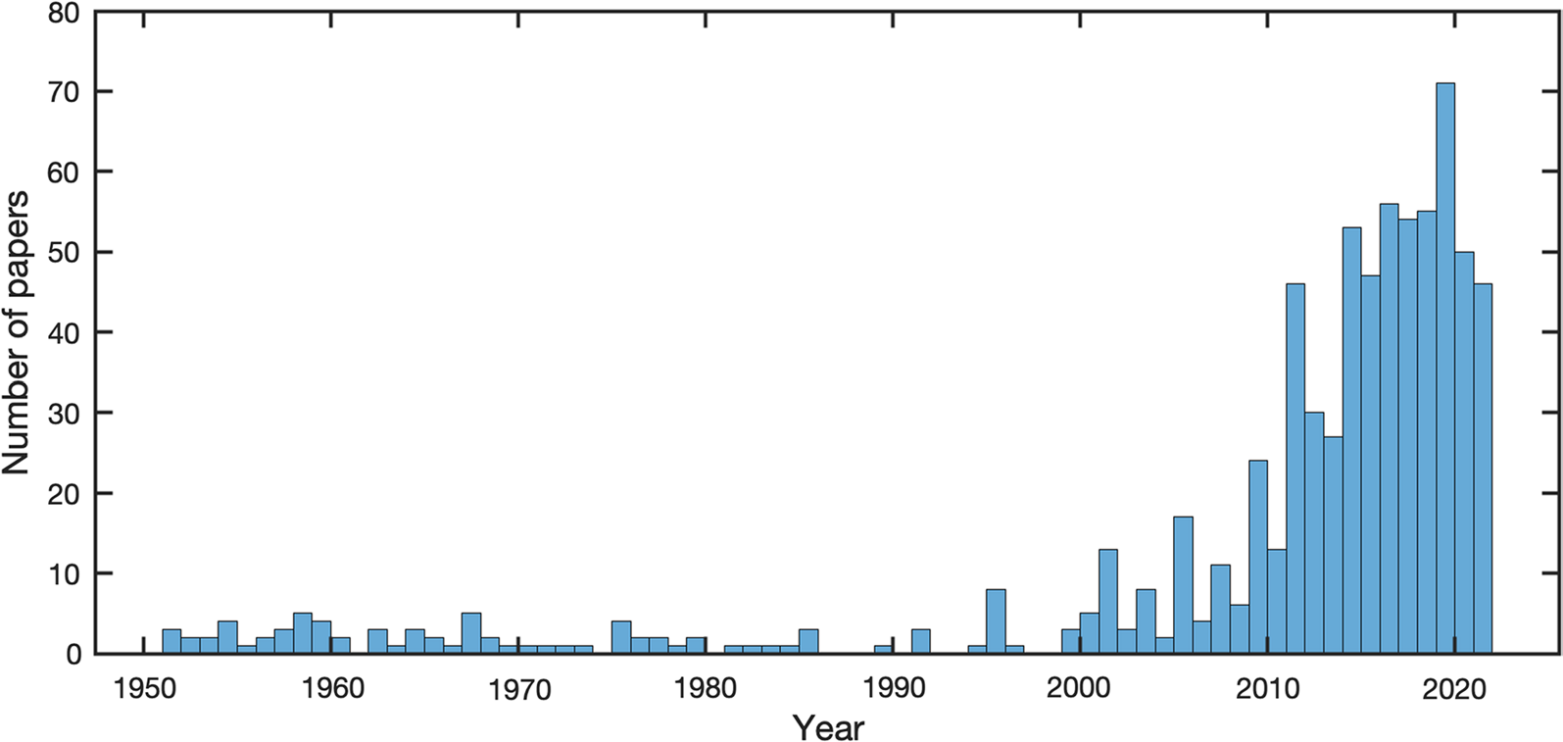
Number of papers published per year from 1951 to 2022 including the term “prosthetic embodiment” in the title or keywords. Data from CrossRef, 18th January 2022

A remarkable amount of recently published work on the advances of prosthetic technology has linked improvements in control and sensory feedback to an increased sense by the users to perceive the artificial limb as embodied (e.g. [1–4]). Consequently, the concept of embodiment has grown in popularity over the years (see Fig. 1), arguably under the assumption that this is an important factor for successful prosthesis adoption (e.g. see [5–8] for various formulations of this hypothesis).

As we have shown in an accompanying review to this article (see summary box), embodiment is a hazy notion often undefined or defined implicitly [9]. If the ambition is to use prosthetic embodiment as a metric of success with practical implications for translational research, the field needs to converge not only on its definition but also on the experimental framework to investigate it. The work conducted in our parallel review [9], which extends to the current article, aimed to facilitate this convergence.

Brief summary of the accompanying systematic review [9] A thematic analysis revealed that prosthetic embodiment can be conceptualized in terms of **body representations** (i.e., “the prosthesis becomes part of a body representation”) or **phenomenology** (i.e., “the prosthesis is perceived as part of the body”). We concluded that prosthetic embodiment is preferably conceptualized as an experimental phenomenological construct that combines **ownership and agency** and thus renders it into a quantifiable metric that can be used in translational research.

We further summarized different ownership and agency experiments and provided a list of **explicit ownership** (e.g., questionnaires), **implicit ownership** (e.g., proprioceptive drift, galvanic skin response, and normalization of phantom limb length), **explicit agency** (e.g., questionnaires), and **implicit agency** (e.g., intentional binding) measures as a reference to be used in prosthetic embodiment studies.

It is worthy of notice that even after agreeing on a common definition and standardized ways to investigate embodiment, further considerations are still required. Namely, it is essential to also consider the properties and principles that govern prosthetic embodiment to ensure that results found in the laboratory are applicable in everyday life environments, which is the end goal of prosthetic research.

For example, in our review of the definitions of embodiment, we found that the concept is oftentimes oversimplified and flattened into a dichotomy where a prosthesis is perceived as either a body part or a tool [9]. The dynamic nature of embodiment is also an aspect often neglected due to the limitations of the experimental paradigms commonly used (e.g., the rubber hand illusion is the most common paradigm adopted for assessing ownership in static settings).

In the present article, we aim to offer an in-depth view of the complexity of embodiment. Specifically, we present a perspective on how ownership and agency arise and concur with the experience of embodiment. We first give an account of what seem to be the necessary conditions that enable the senses of ownership and agency, and then identify the common denominators among the different theories for their emergence. In the discussion, we add a layer of analysis by presenting a multi-dimensional framework of prosthetic embodiment. The framework spans the range of possible interactions of the prosthetic user with the environment and conceptualizes embodiment as existing along a spectrum. The end goal of this work is to provide the context required to better understand prosthetic embodiment research and thereby make its results more relevant and readily applicable to prosthetics outside the research laboratories.

## Perceptual rules of ownership and agency

In our previous systematic review, we conceptualized prosthetic embodiment as an experimental phenomenological combination of ownership and agency, where ownership is the awareness that parts of our body belong to ourselves, and agency is the awareness that we are the initiator of actions.

Observations and conclusions from studies on bodily illusions, such as the Rubber Hand Illusion (RHI), have provided empirical evidence for formulating perceptual rules governing bodily ownership [10–12]. Agency differs from ownership in that it can be felt beyond one’s body and the perceptual constraints for this sense have historically been charted through experiments investigating the feeling of control over external events (e.g. studies on intentional binding). Yet, Wen noted that the perceptual constraints for external agency might be more flexible and adaptable than those for body agency (e.g. with regard to the delays between action and effect in temporal binding paradigms) [13]. This distinction should be considered when reviewing the perceptual rules for agency in the context of prosthetic control, as the line between external- and self-agency is not always drawn clearly in the literature.

Inspired by Ehrsson [12] and Abulkarim [14], in the following section we summarize the perceptual rules for ownership and agency and provide supporting evidence with relevance for prosthetic embodiment (Table 1). Non-adherence to these perceptual rules can decrease or even inhibit the emergence of ownership or agency.

**Table 1:** Perceptual rules for emergence and maintenance of ownership and agency

Ownership
Temporal rule: “Multisensory stimuli need to be synchronous”
Spatial rule: “Sensory inputs need to be spatially congruent and anatomically plausible”
Anatomical rule: “The viewed body part needs to have a humanoid shape”
Tactile congruence rule: “Seen and felt stimuli need to be congruent in terms of tactile properties”
Agency
Volition rule: “Action intent is necessary”
Spatial rule: “The action (or its predicted outcome) and its consequence needs to be spatially congruent.”
Temporal contiguity rule: “The temporal relationship between action and effect needs to be contiguous”

### Ownership

#### Temporal rule: “Multisensory stimuli need to be synchronous”

The feeling of ownership is dependent on the synchronicity of incoming stimuli. RHI studies have shown that discrepancies in the timing of merely 300–400 ms significantly decrease the perceived explicit ownership (measured via a questionnaire) over a rubber hand [15, 16], as well as its implicit ownership (measured via ﻿functional magnetic resonance imaging (fMRI) and proprioceptive drift, respectively).

#### Spatial rule: “Sensory inputs need to be spatially congruent and anatomically plausible

A multitude of circumstances can influence the perceived congruence of stimuli, such as the orientation and location of applied and perceived visual, tactile, and proprioceptive inputs. For example, RHI experiments with able-bodied participants have shown a decrease in explicit ownership (measured via a questionnaire) [17–19] and in implicit ownership (measured via proprioceptive drift) [18] with distances larger than 20–30 cm between the real and the rubber hand. A decrease in explicit ownership (measured via questionnaires) and implicit ownership (measured via proprioceptive drift) has also been reported with rubber hands that are not aligned and/or oriented with the real hand with regard to a hand-centered spatial reference frame [11, 20–22].

#### Anatomical rule: “The viewed body part needs to have a humanoid shape”

Studies have shown that for static objects, explicit and implicit ownership were only reported if the objects were body-shaped [23, 24]. Objects that are not shaped like a human hand have not been found to elicit ownership [21, 23, 25]. Furthermore, non-anatomical color or material, given a hand-shaped object, did not inhibit explicit or implicit ownership. For example, ownership has been shown to arise for non-matching skin color [26], wooden hands [11], and metallic robotic hands [27].

#### Tactile congruence rule: “Seen and felt stimuli need to be congruent in terms of tactile properties”

RHI experiments have shown that incongruencies between the properties of the tools used to touch the rubber and the real hand led to a reduction of the illusion strength. For example, one experiment showed that the discrepancies introduced by touching the rubber hand with a pencil, and the real hand with a paintbrush, were sufficient to significantly reduce the feeling of ownership [28]. The same experiment showed that mere variations in terms of smoothness or roughness (i.e., using a mascara brush vs a paintbrush) did not affect the illusion; however, a more recent study using similar objects differing only in terms of texture demonstrated that even small discrepancies in tactile congruency lead to a significant reduction in ownership [29].

### Agency

#### Volition rule: “Action intent is necessary”

Volition (or intention) has been referred to as a “desired state” that one intends to achieve through action [30]. When it comes to sensorimotor control, the comparator model is well-supported by empirical evidence [31], which proposes that the sense of agency requires motor volition (intentionality for active movement) [32]. Studies using passive movement paradigms show that, without active movements, the sense of agency (as measured by self-report) is either absent [11] or weakened (as measured via intentional binding) [33, 34]. It is important to note that in passive movement studies, the intentionality of action cannot be entirely ruled out since participants could engage in mental simulation and prediction, activating their motor system [35]. In this regard, a recent study addressed the question of whether intentional binding, as a reflection of the sense of agency, occurs in absence of motor action [36]. The study observed that in absence of motor action, implicit agency is dramatically disrupted.

#### Spatial rule: “The action (or its predicted outcome) and its consequence need to be spatially congruent”

Explicit agency (as measured by self-report) has been shown to decrease when an angular deviation between observed movement and actual movement was present [37]. However, simply increasing the distance between the rubber and the real hand seems not to decrease explicit agency [18]. Further, explicit agency over hand representations seems to only emerge if the correct finger responds to the intended movement and is easily disrupted otherwise [37].

#### Temporal contiguity rule: “The temporal relationship between action and effect needs to be contiguous”

The temporal discrepancy between an action and its effect diminishes the sense of agency [13]. Both large delays and unpredictable delays have been shown to reduce agency. Timing discrepancies of 200 ms between visual feedback and movement lead to the perception of a delay [38, 39]. Consequently, explicit agency (measured via questionnaires) over one’s movement was reported to decrease with increasing delays in sensory feedback [40–42]. However, in another experiment, when given the option to attribute a temporally delayed volitional movement to themselves, or to an external cause, participants chose themselves even when presented with delays up to 1100 ms [43]. It appears that the length of delay sufficient to diminish the sense of agency varies among different circumstances [44], and that individual differences might play a role [42]. It has also been argued that self-reports with a numeric scale rating are not sensitive enough for an accurate measure of agency [43].

### Multisensory integration and volition

Various neurocognitive models for ownership and agency have been proposed through the years. An excellent overview of these models can be found in Braun et al*.* [45]. Using their terminology, models for ownership can be placed in a continuum between top-down and bottom-up accounts. Top–down theories put a strong emphasis on relevant body representations, while bottom-up theories rely more heavily on multisensory integration processes to explain the emergence of ownership. Regardless, all these models are theorized around the mechanisms of integration of visual, tactile, and proprioceptive signals and provide a neuroscientific understanding of the temporal, spatial, and other congruency constraints described earlier, which align well with the congruency principles of multisensory integration [12]. In this perspective, ownership can be regarded as a coherent multisensory perception of one’s own body [12].

In a recent review, Wen and Imamizu [30] highlight the three key aspects of agency—intention, effect, and action, and summarize the main models used to explain the emergence of agency. While the processing of actions and effects are directly linked to the motor and perceptual systems, the detection and self-attribution of action-effect contingencies are inextricably linked by action intentionality (volition). Three main theories for agency can be identified: the comparator model, the theory of apparent mental causation, and the Bayesian integration models [30, 46–51]. The commonality between these models is that they explain the emergence of agency in terms of the mechanisms underlying the integration of a large variety of internal and external cues.

One important consideration when studying ownership and agency of prosthetic limbs is that they are rarely experienced in isolation. A few experiments have explored the question of whether agency and ownership are dissociated and simply co-occur, or if there exists an interplay between them [18, 52–58]. Braun et al*.* reviewed the findings of these experiments and concluded that, although they can be partially double dissociated by carefully tweaking the experimental settings in an artificial setup, if agency and ownership co-occur, they strengthen each other [45]. This is most likely the situation when considering a prosthetic limb used in real life.

In summary, ownership and agency are governed by the mechanisms underlying the integration of cues coming from multisensory channels of information and, in the case of agency, internal volitional information [59–62].

## Discussion

### Ownership and agency in prosthetics

Most studies on the emergence of ownership and agency were conducted with able-bodied participants. However, recent studies showed that the perceptual rules for ownership and agency apply also to people with amputation [63–65]. Nevertheless, key differences between how these perceptual constraints apply to a prosthesis compared to a biological hand are worthy of pointing out.

The same temporal constraints hold for feeling ownership over a prosthesis as were discovered with the RHI experiment: visual and somatosensory stimuli need to be perceived nearly simultaneous for explicit ownership (measured via questionnaires) and implicit ownership (measured via proprioceptive drift, skin conductance response, and normalization of phantom-limb length) to arise [3, 64, 65]). While the influence of temporality on individual actions in able-bodied experiments is still debated, delays between movement intent and executed prosthetic movement are argued to decrease agency. Wen reasoned that, in tasks where continuous outcome and intent comparisons are present, delay leads to prosthesis users failing to recognize the effects caused by their own actions [13]. She argued that “if the delay between action and feedback is long, and too many motor commands have been executed before the input of corresponding feedbacks, the memory buffer may ‘overflow’, resulting in failures of comparisons and loss of sense of agency” ([30], p. 4).

Further, there are no spatial discrepancies in the traditional sense: a properly-fitted prosthesis spatially aligns with the trunk and the residual limb like a biological limb would align. However, for people experiencing a frozen [66] or telescoped [67] phantom limb, a significant spatial discrepancy can arise between the prosthesis and the perceived phantom limb—potentially leading to a decrease in both ownership and agency.

### The multi-dimensional prosthetic embodiment spectrum

**Fig. 2:**
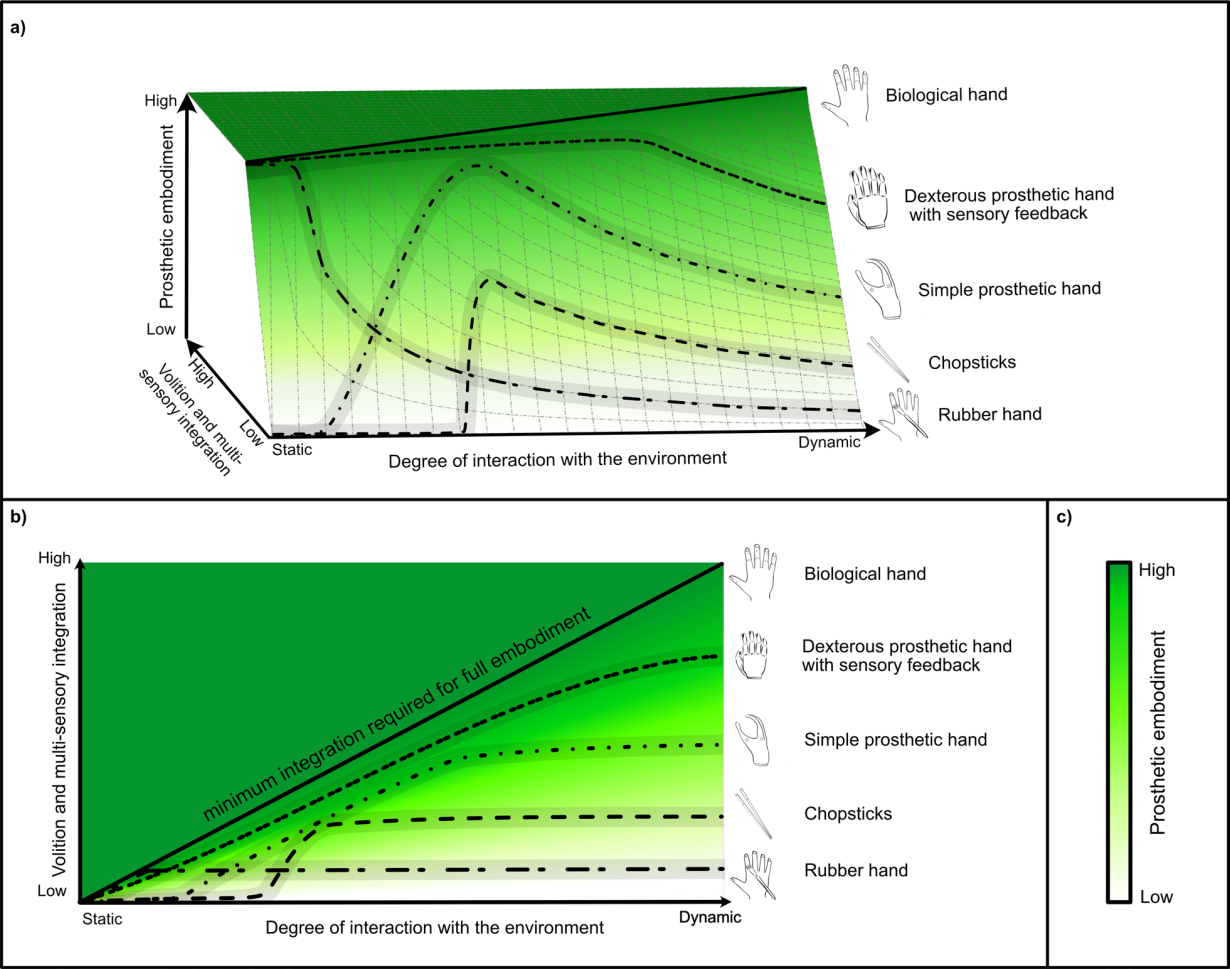
Representations of the multi-dimensional prosthetic embodiment spectrum in which a biological hand demarks the embodiment horizon. **a** 3D representation of prosthetic embodiment depending on the degree of interaction with the environment and the degree of integration of volition and multi-sensory information. A static object (e.g., a rubber hand) requires few sensory modalities to integrate correctly for embodiment to arise (e.g., visuo-tactile congruency). On the other side of the spectrum, an object dynamically interacting with the environment (e.g., a dexterous prosthetic hand) needs both the volition and all multi-sensory inputs to integrate correctly for embodiment to arise. Between the spectrum endpoints, we find chopsticks as a representative tool as an example, which due to their limited sensory feedback does not fulfill the sensory integration criteria for embodiment in a static environment. In a more dynamic environment, however, movements of a tool arising from volition can contribute to a partial, yet bounded embodiment experience. The grey bands indicate personal and circumstantial factors that can modulate the perceived prosthetic embodiment. **b** 2D projection of the multi-dimensional spectrum of panel **a** for more compact visualization. **c** The ideal one-dimensional embodiment scale represents fully dynamic interaction with the environment, which is arguably the ideal condition of operation for prosthetics

Based on our own clinical observations [68] and the above-discussed theories and principles of ownership and agency emergence (see Fig. 2), we propose a multi-dimensional framework on how prosthetic embodiment, as defined by the combination of ownership and agency [9], can be considered in translational research.

Agency and ownership themselves emerge from the successful integration of internal and external cues on sensory input coming from a multitude of different channels, and internal predictions made based on motor volition. Before delving into the details of the framework, it is important to point out that this is not intended as an all-encompassing theory for the emergence of prosthetic embodiment. For instance, we deliberately do not address the precise mechanism of integration, or the weight of agency and ownership to create embodiment and if one is more relevant than the other. Rather, we aim to provide a framework that encompasses the different theories and in which translational research can be conducted.

The multi-dimensional framework of prosthetic embodiment emphasizes the necessity of multisensory and volition integration among spatiotemporally coincident stimuli (Y-axis in Fig. 2). When correctly integrated, both in terms of “multisensory integration” [69] and of motor volition [32, 70], the multimodal input affords a certain degree of prosthetic embodiment. The multi-dimensional framework further distinguishes between different degrees of interaction with the environment: here the X-axis in Fig. 2 should be interpreted as the level of interaction that is demanded of the subject in the study. For example, the RHI in static conditions is created by synchronous visuotactile stimulation: in such a circumstance, the conditions necessary for reaching full embodiment are met and therefore the rubber hand is reported to be perceived as the real hand. However, moving towards the right-hand side of the spectrum introduces higher demands on the multimodal input that must be provided to maintain the same level of embodiment (Z-axis in Fig. 2), and this is due to the increasing degree of interaction with the environment. In a dynamic environment, a static rubber hand is no longer fully embodied as a non-controllable object cannot meet the now raised demands of interaction. Conversely, in a restricted dynamic environment, even a tool such as chopsticks can be partially embodied as it is afforded by the emergence of agency. Lastly, in an environment with high demands of dynamic interaction, both ownership and agency are necessary for full embodiment as is the case with a biological hand (ownership and agency within the whole spectrum of possible static and dynamic interactions with the environment). In Fig. 2, the biological hand demarks the horizon for full embodiment through the multi-dimensional spectrum.

In a fully-dynamic environment (e.g., when performing any instrumental activity of daily living; far right-hand side of the spectrum in panels *a* and *b* of Fig. 2), only partial prosthetic embodiment might be possible by a conventional myoelectric hand. Under perfect operational conditions (e.g., no misclassification error during use), only limited sensory feedback and a limited number of degrees of freedom are available. In comparison, a more dexterous prosthetic hand with sensors to measure the interaction with its environment could allow for (a) an increased sense of agency by allowing more degrees of freedom to be controlled, and (b) an increased sense of ownership due to additional sensory feedback, possibly yielding a higher level of embodiment.

A prosthetic limb should ideally substitute for the biological one allowing for interaction with the world without additional limitations. In this ideal situation, the three-dimensional prosthetic embodiment representation could be simplified to the state of full dynamic interaction with the environment, and thus be reduced to an *ideal one-dimension embodiment scale* (panel *c* of Fig. 2). In the ideal one-dimension scale, the level of volition and multi-sensory integration (combined agency and ownership) is synonymous with embodiment in a fully dynamic environment. However, prosthetic technology is far from reaching such an ideal situation, and therefore we foresee that the interaction spectrum will continue to be relevant in the coming years.

**Fig. 3:**
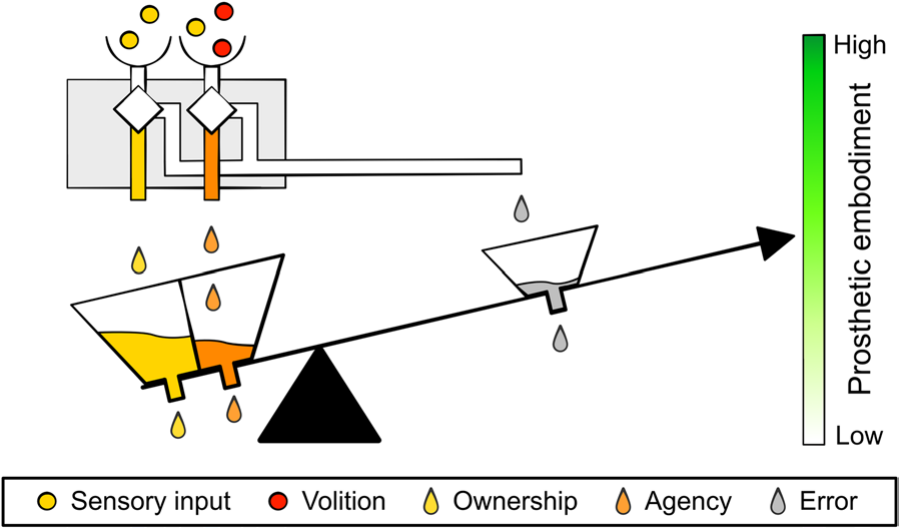
Prosthetic embodiment scale: multi-sensory input and volition are evaluated in an integration process. If the integration is successful, ownership and agency arise, and embodiment is experienced. In this analogy, the successful integration of volition (red circles) and multi-sensory input (yellow circles) is visualized by the creation of water droplets and the mixing of color in the case of agency. Both ownership and agency droplets increase the weight of their respective tanks, thus contributing to prosthetic embodiment (as indicated by the lever). Full prosthetic embodiment can only be reached by experiencing both ownership and agency. Integration discrepancies (grey droplets) do not lead to ownership and/or agency and fill the tank on the other arm of the leaver reducing embodiment (the tank on the opposite side increases weight as more droplets fail to go through the integration process). Droplets leaking from the reservoirs represent a loss of accumulated ownership, agency, and error over time. The location of the ownership and agency tanks with respect to each other was done arbitrarily in the figure that is meant to serve solely as a visual analogy

An implication of the multi-dimensional framework of embodiment is that the experimental findings from single points along the interaction spectrum (static to dynamic, X-axis in Fig. 2) can be arranged hierarchically according to the value they hold for translation into daily life usage. The finding that a prosthesis is successfully embodied through the RHI paradigm (e.g., references [3, 64, 65]), although of great scientific interest, does not necessarily predict whether the prosthesis will be perceived as embodied when interacting with the environment. Conversely, experiments assessing embodiment with functional tests and a more dynamic interaction with the environment (e.g., the dynamic Prosthesis Incorporation (PIC) assessment based on the cross-model congruency paradigm implicitly measuring ownership [71, 72]) should be given a higher place in the hierarchy of embodiment relevance. Alternatively, a penalty or cap on the ideal one-dimension embodiment scale could be introduced to prevent experiments on limited dynamic interactions to reach the full magnitude of embodiment. In Fig. 3, we break down this concept further with an analogy where the weighted sum of ownership and agency on a lever indicates the current prosthetic embodiment. Because detailed roles of and interactions between ownership and agency are still researched, equal relative weighting was assigned to ownership and agency.

The one-dimensional prosthetic embodiment scale for example depicts that experiencing only ownership, as in the case of the RHI, or mostly agency, as in the case of the chopsticks, is not sufficient to move the lever up to the highest level of prosthetic embodiment. An important feature of the analogy in Fig. 3 is the explicit depiction of the dependency of embodiment on time—a dimension previously embedded within the dynamic spectrum in Fig. 2.

An example of the dependency on volition and multisensory integration is when the multi-sensory integration fails (e.g., when the prosthesis breaks down or functions sub-optimally) instantly disrupting the sense of belonging to the body [68]. The temporal aspect of multi-sensory interaction affects prosthetic embodiment in general and over multiple timescales: from an hourly basis, as the user dons and doffs the prosthesis, to longer periods reflecting the effects of training and learning on prosthetic embodiment. It has been shown that sufficiently prolonged training seems to lead to more pre-reflective and natural use [73] (likely owing to the development of a distinct forward model adapted to the specific prosthesis) and a cross-modal representation of the artificial limb akin to real limbs [74].

Individual experiences of body ownership and agency have been widely documented in the literature. For example, it has been observed that roughly 20–30% of the population do not experience body ownership during the RHI [17, 75, 76]. Research into individual differences in susceptibility to the RHI has shown that this could depend on interoceptive sensitivity [77] or sensory suggestibility [78]. Other research has instead suggested that agency is modulated by mental state [79], task performance [80], and social interactions [81]. One important limitation of the framework proposed here is that, as for many models for agency and ownership, there is no account for how individual factors, prior expectations, and experience can influence the current state of embodiment. The modulatory power of such individual factors can be imagined as an additional dimension of this framework, resulting in an additional axis. Notably, probabilistic models for multisensory perception for ownership [60, 82] and the Bayesian integration models for agency [50, 51] include prior beliefs in the processes that give rise to these experiences.

## Body part or tool: a false dichotomy

A recurring theme in the prosthetic literature is to describe embodiment of a prosthesis in terms of being perceived as a body part as opposed to a tool (e.g., references [5, 8, 83–86]). Within the proposed two-dimensional prosthetic embodiment spectrum, however, tools can also be embodied and lie on the low end of the prosthetic embodiment scale. Interestingly, even a biological arm has been described as a tool by prosthesis users [68]. Distinguishing between tools and prostheses when claiming embodiment is not a trivial task; for example, current implicit measures used for prosthetic embodiment might not actually discriminate between the embodiment of handheld objects (such as mobile phones) that can be perceived as a body extension and rubber hands [87]. Ensuring that a prosthesis achieves higher embodiment than a tool would entail paradigms that put the two in direct comparison, showing that the prosthesis achieves higher levels of agency and ownership. We suggest that such an approach should be followed in general, not only when trying to show that the prosthesis is not embodied as a body part. Specifically, many nerve stimulation studies have evaluated embodiment with the RHI paradigm with the asynchronous stimulation condition as control (e.g., [2, 3, 88]). We argue that an additional useful comparison would include another device (a tool) that is deemed to yield lower embodiment.

## Allure of embodiment to justify sensory feedback research

Another recurring theme in the prosthetic literature is the claim that a lack of sensory feedback is the leading factor behind prosthesis abandonment [89, 90]. Similarly, embodiment itself has been claimed as crucial for prosthesis acceptance [8, 91–93]. However, no evidence has been provided to substantiate that sensory feedback or embodiment is required for prosthetic acceptance, nor that the lack thereof is a cause for prosthetic abandonment.

Although sensory feedback has indeed emerged in some surveys as one of the factors impacting acceptance [94, 95], it is not the factor with the highest priority [96, 97]. For instance, it is not spontaneously mentioned as something important [98]. User surveys have instead consistently highlighted functionality [99, 100], reliability [101, 102], better control mechanisms [103], ability to provide increased dexterity [98], or weight [96, 102] as important user needs. One opposing view to this could be that sensory feedback has the potential to affect and improve all the above-mentioned factors. For example, it has been shown that sensory feedback via neural stimulation decreases the perceived weight of the prosthesis [104]. Yet, given that the effects of sensory restoration via electrical stimulation are secondary or indirect, a possibly better way to solve the prosthesis rejection problem would be to address directly what has been highlighted as important by user surveys. In the case of weight, it would be to reduce the actual weight of the prosthesis, as well as to solve the mechanical attachment problem because it is the compression exerted by conventional sockets which makes weight a bigger problem.

It is also important to remark that other forms of sensory feedback beyond direct neural stimulation are commonly provided by artificial limbs. In absence of somatosensory feedback, motor control relies on alternative sensing modalities, such as vision, audition, and vibration [105, 106]. For myoelectric devices, information related to the state of the motors is transferred to the residual limb as vibration or pressure, in addition to auditory feedback (i.e., the hum of the motors). It has been shown that amputees can exploit this type of feedback [107], for example, to estimate prosthesis closing velocity [108]. Direct skeletal attachment of the prosthesis provides additional feedback through osseoperception via tactile and auditory pathways [109]. Body-powered prostheses are known to supply indirect proprioceptive feedback thanks to the fixed relationship between cables and joint angles [110]. This incidental sensory feedback could explain why training and adaptation might play an important role in achieving embodiment [73] and why embodiment seems achievable with various types of prostheses regardless of whether they are body-powered or myoelectric [8], functional or cosmetic [111], or when directly fixated to the bone via osseointegration with [112, 113] or without peripheral nerve stimulation [114, 115]. These findings reiterate the importance of having reliable means to quantify the level of embodiment that a certain prosthesis can achieve.

## Conclusions

In this article, we offer a review of the alleged principles for the emergence of ownership and agency, which combined give rise to prosthetic embodiment. Based on these principles and current clinical evidence, we propose a multi-dimensional framework for contextualizing embodiment research—research that aims at developing prosthetic devices transparent and more useful to the user in daily life.

An advanced prosthetic limb should ideally substitute a biological one and allow full interaction with the world, therefore testing whether a prosthesis is embodied should consider these requirements. This translates into a prosthetic embodiment scale, where both agency and ownership need to emerge and concur to experience embodiment. In this embodiment scale, prosthetic embodiment does not automatically mean that a prosthesis is perceived as a body part. Prostheses that are perceived as a tool, but are functional and grant some degree of ownership and/or agency, can still yield embodiment, albeit to a limited extent.

Another important point raised by our analysis of the literature is that prosthetic devices offer different channels of incidental feedback via alternative sensing modalities such as vision, audition, and vibration, all of which can contribute to volition and multisensory integration required for embodiment. Therefore, utmost caution is advised in claims that frame sensory feedback provided by electrical stimulation as a necessity to achieve prosthetic embodiment and as the solution to the prosthetic abandonment problem. As shown, several factors are involved in prosthesis user satisfaction, and thus increasing prosthesis acceptance will likely require improving several of them, above all functionality. This, in turn, will lead to increased prosthetic embodiment.

## Data Availability

Not applicable.
